# Supplementary material for: Human Health Risk Assessment Is Associated with the Consumption of Metal-Contaminated Groundwater around the Marituba Landfill, Amazonia, Brazil
Source: Int J Environ Res Public Health. 2022 Oct 25;19(21):13865. doi: 10.3390/ijerph192113865 (PMC9656318; doi:10.3390/ijerph192113865)
Supplement: Supplementary file 1 [file ijerph-19-13865-s001.zip › ijerph-1847186-supplementary-SI.pdf]

International Journal of
Environmental Research and Public Health

MDPI

Table S1. Geographical location and depth of groundwater wells around the landfill site, Amazonia, Brazil.

Point	Geographical Coordinates		Source of Supply	Depth	Neighbourhood
**P01**	01°23′49.4″	048°20′49.8″	Artesian well	17	São João
**P02**	01°23′50.9″	048°20′45.6″	Artesian well	18	São João
**P03**	01°23′50.0″	048°20′48.8″	Amazon well	15	São João
**P04**	01°23′47.8″	048°20′45.0″	Amazon well	6	São João
**P05**	01°23′41.5″	048°20′49.2″	Amazon well	12	São João
**P06**	01°23′39.1″	48°20′52.4″	Artesian well	18	São João
**P08**	01°23′23.9″	048°20′55.5″	Artesian well	18	São João
**P09**	01°23′24.2″	048°20′58.2″	Artesian well	18	São João
**P10**	01°23′30.0″	048°20′57.6″	Artesian well	18	São João
**P11**	01°23′17.6″	048°20′51.9″	Artesian well	18	Uriboca
**P12**	01°23′15.8″	048°20′50.8″	Artesian well	18	Uriboca
**P13**	01°23′12.6″	048°20′51.1″	Amazon well	18	Uriboca
**P14**	01°23′12.0″	048°20′50.9″	Artesian well	13	São João
**P15**	01°23′11.8″	048°20′48.7″	Artesian well	18	São João
**P16**	01°23′14.1″	048°20′46.7″	Artesian well	18	Uriboca
**P17**	01°23′16.4″	048°20′44.2″	Artesian well	18	Uriboca
**P18**	01°23′16.3″	048°20′46.0″	Artesian well	17	Uriboca
**P19**	01°23′15.3″	048°20′41.7″	Amazon well	17	Uriboca
**P20**	01°23′14.8″	048°20′38.7″	Amazon well	12	Uriboca
**P21**	01°23′15.1″	048°20′33.8″	Amazon well	12	Uriboca
**P22**	01°23′08.1″	048°20′22.4″	Artesian well	14	Santa Lúcia I
**P23**	01°23′11.1″	048°20′33.9″	Artesian well	22	Uriboca
**P24**	01°23′12.5″	048°20′55.3″	Artesian well	23	São João
**P25**	01°23′12.2″	048°21′00.0″	Artesian well	24	São João
**P26**	01°23′10.6″	048°21′00.4″	Artesian well	12	São João
**P27**	01°23′10.3″	048°20′59.5″	Artesian well	12	São João
**P28**	01°23′12.5″	048°20′55.2″	Artesian well	18	São João
**P29**	01°23′12.2″	048°20′54.6″	Artesian well	18	São João
**P30**	01°23′12.2″	048°20′52.54″	Artesian well	12	São João
**P31**	01°23′11.7″	048°23′11.7″	Amazon well	18	São João
**P32**	01°23′10.4″	048°20′50.4″	Amazon well	6	São João
**P33**	1°23′10.36″	048°20′50.22″	Artesian well	16	São João
**P34**	01°23′18.4″	048°20′34.6″	Artesian well	12	Uriboca
**P35**	01°23′20.2″	048°20′33.7″	Amazon well	15	Uriboca
**P36**	01°23′20.5″	048°20′35.2″	Amazon well	10,5	Uriboca
**P37**	01°23′15.8″	048°20′32.8″	Artesian well	9	Uriboca
**P38**	01°23′15,1″	048°20′27.4″	Artesian well	17	Uriboca
**P39**	01°23′13.4″	048°20′25.5″	Artesian well	17	Uriboca
**P40**	01°23′10.8″	048°20′26.5″	Artesian well	17	Uriboca
**P41**	01°23′09.4″	048°20′28.1″	Artesian well	18	Uriboca
**P42**	01°23′09.5″	048°20′33.2″	Artesian well	18	Uriboca
**P43**	01°23′12.6″	048°20′35.3″	Amazon well	13	Uriboca
**P44**	01°23′06.8″	048°20′14.1″	Artesian well	17	Beira Rio
**P45**	01°23′08.5″	048°20′15.3″	Artesian well	18	Beira Rio
**P46**	01°23′08.7″	048°20′18.0″	Artesian well	24	Beira Rio

Point	Geographical Coordinates		Source of Supply	Depth	Neighbourhood
**P47**	01°23′10.6″	048°20′22.1″	Artesian well	18	Beira Rio
**P48**	01°23′10.3″	048°20′22.0″	Artesian well	18	Beira Rio
**P49**	01°23′09.9″	048°20′18.7″	Artesian well	17	Beira Rio
**P50**	01°23′10.8″	048°20′17.7″	Artesian well	17	Beira Rio
**P51**	01°23′11.5″	048°20′16.3″	Artesian well	18	Beira Rio
**P52**	01°23′12.1″	048°20′13.8″	Artesian well	18	Beira Rio
**P53**	01°23′12.8″	048°20′15.1″	Artesian well	17	Beira Rio
**P54**	01°23′14.3″	048°20′18.0″	Artesian well	18	Beira Rio
**P55**	01°23′15.4″	048°20′16.9″	Artesian well	18	Beira Rio
**P56**	01°23′11.3″	048°20′21.1″	Artesian well	18	Beira Rio
**P57**	01°23′16.8″	048°20′15.8″	Artesian well	18	Beira Rio
**P58**	01°23′15.8″	048°20′14.3″	Artesian well	18	Beira Rio
**P59**	01°23′18.8″	048°20′14.3″	Artesian well	24	Beira Rio
**P60**	01°23′05.4″	048°20′12.3″	Artesian well	24	Beira Rio
**P61**	01°23′05.5″	048°20′12.2″	Amazon well	24	Beira Rio
**P62**	01°23′05.5″	048°20′12.2″	Artesian well	18	Beira Rio
**P63**	01°23′06.7″	048°20′17.1″	Artesian well	18	Beira Rio
**P64**	01°23′05.9″	048°20′17.7″	Amazon well	15	Beira Rio
**P65**	01°23′07.1″	048°20′19.6″	Artesian well	18	Beira Rio
**P66**	01°23′12.6″	048°20′18.8″	Artesian well	30	Beira Rio
**P67**	01°23′14.6″	048°20′13.8″	Artesian well	18	Beira Rio
**P68**	01°23′13.1″	048°20′14.4″	Artesian well	24	Beira Rio
**P69**	01°23′27.3″	048°19′55.0″	Artesian well	18	Santa Lúcia I
**P70**	01°23′28.1″	048°20′03.8″	Artesian well	40	Santa Lúcia I
**P71**	01°23′28.1″	048°20′02.1″	Artesian well	40	Santa Lúcia I
**P72**	01°23′28.2″	048°20′04.0″	Artesian well	40	Santa Lúcia I
**P73**	01°23′27.4″	048°20′04.0″	Artesian well	40	Santa Lúcia I
**P74**	01°23′27.8″	048°20′02.6″	Artesian well	40	Santa Lúcia I
**P75**	01°23′27.7	048°20′02.2″	Artesian well	40	Santa Lúcia I
**P76**	01°23′26.9″	048°20′00.7″	Artesian well	40	Santa Lúcia I
**P77**	01°23′28.1″	048°19′58.0″	Artesian well	18	Santa Lúcia I
**P78**	01°23′27.3″	048°19′57.7″	Artesian well	30	Santa Lúcia I
**P79**	01°23′26.3″	048°19′56.7″	Artesian well	40	Santa Lúcia I
**P80**	01°23′24.9″	048°19′52.4″	Artesian well	40	Santa Lúcia I
**P81**	01°23′24.1″	048°19′56.0″	Artesian well	40	Santa Lúcia I
**P82**	01°23′24.3″	048°19′57.7″	Artesian well	40	Santa Lúcia I
**P83**	01°23′23.3″	048°20′00.4″	Artesian well	40	Santa Lúcia I
**P84**	01°23′25.3″	048°20′02.0″	Artesian well	40	Santa Lúcia I
**P85**	01°23′26.58″	048°20′4.91″	Artesian well	18	Santa Lúcia I
**P86**	01°23′24.21″	048°19′55.00″	Artesian well	40	Santa Lúcia I
**P87**	01°23′27.01″	048°19′56.68″	Artesian well	40	Santa Lúcia I
**P88**	01°23′26.36″	048°19′55.01″	Artesian well	40	Santa Lúcia I
**P89**	01°23′14.2″	048°18′54.0″	Artesian well	40	Decouville
**P90**	01°23′14.5″	048°18′55.1″	Artesian well	266	Decouville
**P91**	01°23′12.7″	048°18′54.6″	Artesian well	266	Decouville
**P92**	01°23′12.7″	048°18′54.1″	Artesian well	266	Decouville
**P93**	01°23′11.9″	048°18′53.9″	Artesian well	266	Decouville
**P94**	01°23′10.9″	048°18′52.6″	Artesian well	266	Decouville

Point	Geographical Coordinates		Source of Supply	Depth	Neighbourhood
**P95**	01°23′09.6″	048°18′53.3″	Artesian well	266	Decouville
**P96**	01°23′10.1″	048°18′53.4″	Artesian well	266	Decouville
**P97**	01°23′10.2″	048°18′53.9″	Artesian well	266	Decouville
**P98**	01°23′11.01″	048°18′55.35″	Artesian well	266	Decouville
**P99**	01°23′10.3″	048°18′56.6″	Artesian well	266	Decouville
**P100**	01°23′10.0″	048°18′55.3″	Artesian well	266	Decouville
**P101**	01°23′09.6″	048°18′56.1″	Artesian well	266	Decouville
**P102**	01°23′7.27″	048°18′55.08″	Artesian well	266	Decouville
**P103**	01°23′08.6″	048°18′56.3″	Artesian well	266	Decouville
**P104**	01°23′7.77″	048°18′55.02″	Artesian well	266	Decouville
**P105**	01°23′07.8″	048°18′58.5″	Artesian well	266	Decouville
**P106**	01°23′05.7″	048°18′55.8″	Artesian well	266	Decouville
**P107**	01°23′7.30″	048°18′59.24″	Artesian well	266	Decouville
**P108**	01°23′06.7″	048°18′58.3″	Artesian well	266	Decouville
**P109**	01°23′05.8″	048°19′00.6″	Artesian well	266	Decouville
**P110**	01°23′04.8″	048°18′58.0″	Artesian well	266	Decouville
**P111**	01°23′04.7″	048°18′58.0″	Artesian well	266	Decouville
**P112**	01°23′02.4″	048°18′58.6″	Artesian well	266	Decouville
**P113**	01°23′02.9″	048°18′58.2″	Artesian well	266	Decouville
**P114**	01°23′4.69″	048°19′0.71″	Artesian well	266	Decouville
**P115**	01°23′04.7″	048°19′00.1″	Artesian well	266	Decouville
**P116**	01°23′03.3″	048°19′01.8″	Artesian well	266	Decouville
**P117**	01°22′59.41″	048°19′3.28″	Artesian well	266	Decouville
**P118**	01°22′55.9″	048°19′03.7″	Artesian well	266	Decouville
**P119**	01°22′56.8″	048°19′04.5″	Artesian well	266	Decouville
**P120**	01°22′56.9″	048°19′04.1″	Artesian well	266	Decouville
**P121**	01°22′57.3″	048°19′05.2″	Artesian well	266	Decouville
**P122**	01°22′56.9″	048°19′06.0″	Artesian well	266	Decouville
**P123**	01°22′58.2″	048°19′05.3″	Artesian well	266	Decouville
**P124**	01°22′58.01″	048°19′6.63″	Artesian well	266	Decouville
**P125**	01°22′54.8″	048°19′04.1″	Artesian well	266	Decouville
**P126**	01°22′55.8″	048°19′04.8″	Artesian well	266	Decouville
**P127**	01°23′11.34″	048°18′49.18″	Artesian well	266	Decouville
**P128**	01°23′10.5″	048°18′48.3″	Artesian well	266	Decouville
**P129**	01°23′09.0″	048°18′46.7″	Artesian well	266	Decouville
**P130**	01°23′07.0″	048°18′43.6″	Artesian well	24	São Pedro
**P131**	01°23′06.8″	048°18′45.3″	Artesian well	266	Decouville
**P132**	01°23′05.0″	048°18′43.6″	Artesian well	30	São Pedro
**P133**	01°23′06.7″	048°18′44.4″	Amazon well	10	São Pedro
**P134**	01°23′06.8″	048°18′49.2″	Artesian well	266	Decouville
**P135**	01°23′03.4″	048°18′52.0″	Artesian well	266	Decouville
**P136**	01°23′03.0″	048°18′50.3″	Artesian well	266	Decouville
**P137**	01°22′57.7″	048°18′54.8″	Artesian well	266	Decouville
**P138**	01°23′16.23″	048°18′44.80″	Artesian well	266	Decouville
**P139**	01°23′14.6″	048°18′42.4″	Artesian well	266	Decouville
**P140**	01°23′13.5″	048°18′42.8″	Artesian well	266	Decouville
**P141**	01°23′13.26″	48°18′42.12″	Artesian well	266	Decouville
**P142**	01°23′14.1″	048°18′46.2″	Artesian well	266	Decouville

Point	Geographical Coordinates		Source of Supply	Depth	Neighbourhood
**P143**	01°23′13.3″	048°18′44.3″	Artesian well	266	Decouville
**P144**	01°23′12.2″	048°18′43.0″	Artesian well	266	Decouville
**P145**	01°23′11.3″	048°18′44.9″	Artesian well	266	Decouville
**P146**	01°23′12.5″	048°18′46.1″	Artesian well	266	Decouville
**P147**	01°23′11.6″	048°18′47.2″	Artesian well	266	Decouville
**P148**	01°23′10.2″	048°18′45.1″	Artesian well	266	Decouville
**P149**	01°23′08.1″	048°18′47.2″	Artesian well	266	Decouville
**P150**	01°23′04.5″	048°18′51.0″	Artesian well	266	Decouville
**P151**	01°23′02.5″	048°18′55.0″	Artesian well	266	Decouville
**P152**	01°22′56.06″	048°18′57.13″	Artesian well	266	Decouville
**P153**	01°22′54.0″	048°18′59.4″	Artesian well	266	Decouville
**P154**	01°22′52.81″	048°18′58.32″	Artesian well	266	Decouville
**P155**	01°22′50.4″	048°18′57.7″	Artesian well	266	Decouville
**P156**	01°22′52.1″	048°19′00.3″	Artesian well	266	Decouville
**P157**	01°22′52.0″	048°19′01.9″	Artesian well	266	Decouville
**P158**	01°22′51.1″	048°19′01.0″	Artesian well	266	Decouville
**P159**	01°22′49.9″	048°19′00.5″	Artesian well	266	Decouville
**P160**	01°22′49.5″	048°18′59.7″	Artesian well	266	Decouville
**P161**	01°22′50.0″	048°19′03.6″	Artesian well	266	Decouville
**P162**	01°22′46.8″	048°19′02.6″	Artesian well	266	Decouville
**P163**	01°22′48.0″	048°19′04.0″	Artesian well	266	Decouville
**P164**	01°22′47.3″	048°19′05.6″	Artesian well	266	Decouville
**P165**	01°22′46.5″	048°19′03.6″	Artesian well	266	Decouville
**P166**	01°23′10.2″	048°18′40.4″	Artesian well	10	Decouville
**P167**	01°23′28.8″	048°18′22.9″	Artesian well	20	São Pedro
**P168**	01°23′28.3″	048°18′23.3″	Amazon well	6	São Pedro
**P169**	01°23′27.1″	048°18′24.2″	Artesian well	20	São Pedro
**P170**	01°23′26.6″	048°18′25.3″	Amazon well	12	São Pedro
**P171**	01°23′22.9″	048°18′27.0″	Amazon well	6	São Pedro
**P172**	01°23′14.7″	048°18′32.9″	Amazon well	6	São Pedro
**P173**	01°23′12.6″	048°18′35.2″	Amazon well	18	São Pedro
**P174**	01°23′10.2″	048°18′36.8″	Artesian well	266	São Pedro
**P175**	01°23′03.3″	048°18′41.1″	Artesian well	18	São Pedro
**P176**	01°23′1.35″	048°18′43.74″	Artesian well	12	São Pedro
**P177**	01°23′01.0″	048°18′45.0″	Amazon well	12	São Pedro
**P178**	01°22′59.4″	048°18′45.8″	Amazon well	12	São Pedro
**P179**	01°22′56.9″	048°18′48.0″	Artesian well	24	São Pedro
**P180**	01°22′53.6″	048°18′49.8″	Amazon well	12	São Pedro
**P181**	01°22′48.8″	048°18′52.3″	Amazon well	13	São Pedro
**P182**	01°22′42.3″	048°18′58.0″	Artesian well	266	São Pedro
**P183**	01°22′45.1″	048°18′58.0″	Artesian well	266	São Pedro
**P184**	01°22′49.4″	048°18′55.4″	Artesian well	266	São Pedro
**P185**	01°22′48.3″	048°18′54.3″	Artesian well	266	São Pedro

Note: 185 water samples were collected, however, P07 refers to an igarapé (surface water), and for this reason was excluded from this study. Totaling 184 samples of underground water from artesian and Amazon type wells.

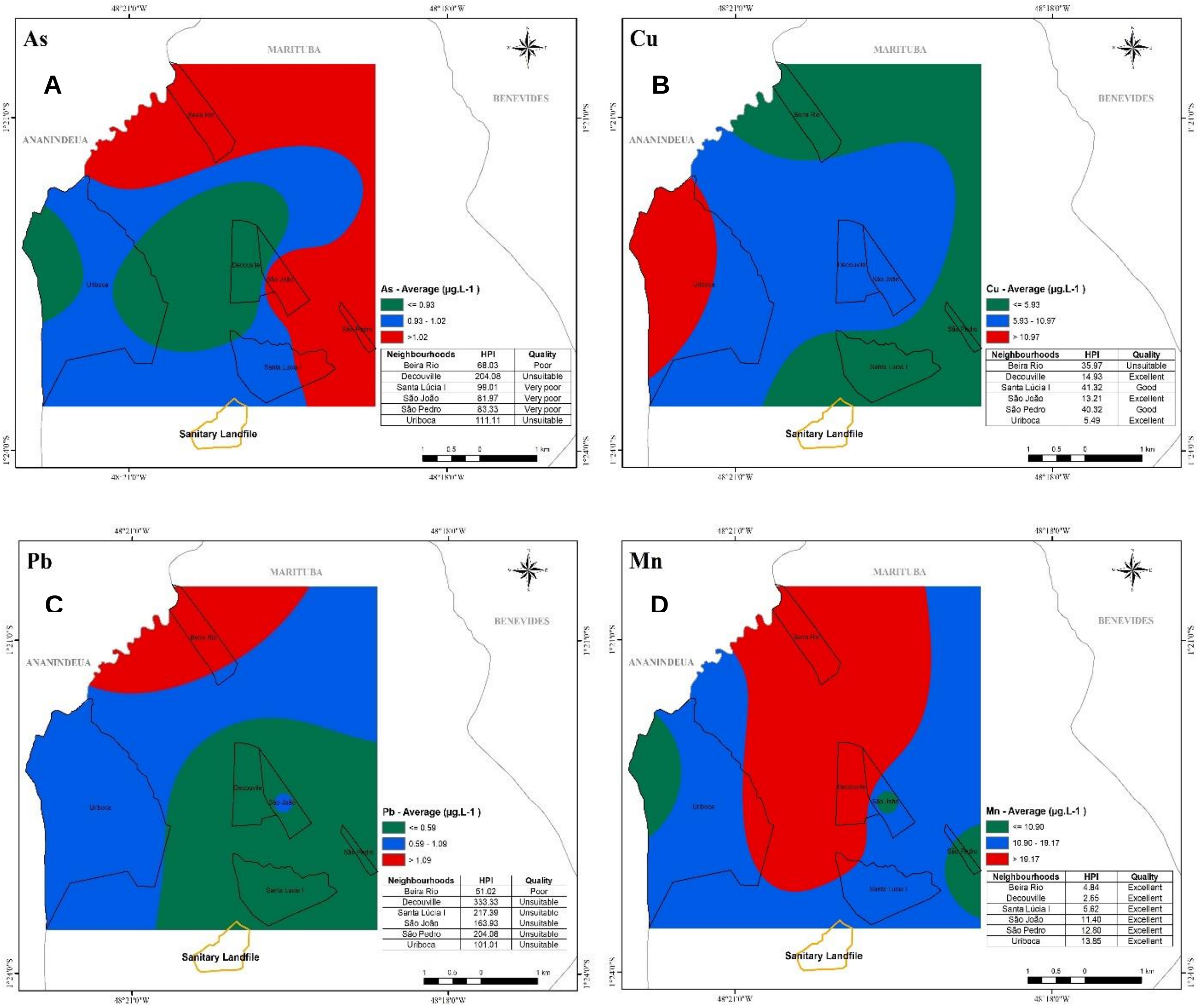

Figure S1. Spatial distribution of mean concentrations for As, Cu, Pb, and Mn and HPI classification in the neighbourhoods surrounding the landfill, Amazonia, Brazil.
